# Infant stimulation reduces weight loss and increases breastfeeding: a randomized controlled trial*

**DOI:** 10.17533/udea.iee.v40n1e05

**Published:** 2022-03-29

**Authors:** Lucy Marcela Vesga Gualdrón, María Mercedes Durán de Villalobos, Nancy Milena Bernal Camargo

**Affiliations:** 1 RN, Ph.D. Assistant Professor. Email: lmvesgag@unal.edu.co Faculty of Nursing, Universidad Nacional de Colombia, Sede Bogotá (Colombia). Universidad Nacional de Colombia Faculty of Nursing Universidad Nacional de Colombia Bogotá Colombia lmvesgag@unal.edu.co; 2 RN, Ph.D. Emeritus Professor. Email: mmvillobos@gmail.com Faculty of Nursing, Universidad Nacional de Colombia, Sede Bogotá (Colombia). Universidad Nacional de Colombia Faculty of Nursing Universidad Nacional de Colombia Bogotá Colombia mmvillobos@gmail.com; 3 MD, Pediatrician. Assistant Professor, Faculty of Medicine, Fundación Universitaria de Ciencias de la Salud, Colombia. Faculty of Medicine Fundación Universitaria de Ciencias de la Salud Colombia

**Keywords:** breast feeding, physical stimulation, anthropometry, mother-child relations, infant, newborn, neonatal nursing., lactancia maternal, estimulación física, antropometría, relaciones madre-hijo, recién nacido, enfermería neonatal., aleitamento materno, estimulação física, antropometria, relações mãe-filho, recém-nascido, enfermagem neonatal.

## Abstract

**Objective.:**

The study aimed to measure the effect of auditory, tactile, visual, and vestibular (ATVV) stimulation therapy on sucking effectiveness (SE), infant-feeding mode, weight, height, and head circumference (HC) of full-term infants.

**Methods.:**

A single-blinded randomized trial with a sample of 107 mother-child dyads. Inclusion criteria were healthy first-time mothers and full-term infants with no known pathological conditions, weighing between 2500 and 4000 grams, and recommendation of exclusive or predominant breastfeeding. The mothers in the experimental group (EG) received training in ATVV stimulation therapy and provided it from birth (first 24 hours of life) until the end of the follow-ups at week 5. The control group (CG) received only standard care that included education on warning signs and basic guidance on breastfeeding. SE, infant-feeding mode, and neonatal growth were measured at weeks 2 and 5.

**Results.:**

In contrast to CG infants, the EG infants drank 2.02 cc more human milk in one minute of effective breastfeeding (*p*=0.002) at week 2 and 5.51 cc more at week 5 (*p*<0.0001). They showed greater adherence to breastfeeding at week 5 (*p*=0.025) and gained more weight: 8.35 grams/day (*p*=0.009) and 4.19 grams/day (*p*=0.008). HC did not differ between groups, and height difference was statistically significant at week 5 (*p*=0.025).

**Conclusion.:**

ATVV stimulation therapy has a positive effect on neonatal health as it promotes effective sucking and exclusive breastfeeding, reduces weight loss, and improves neonatal growth.

## Resumen

**Objetivos.:**

Medir el efecto de la terapia de estimulación auditiva, táctil, visual y vestibular en la succión eficaz, el modo de alimentación, el peso, talla y perímetro cefálico de los neonatos a término.

**Métodos.:**

Ensayo aleatorizado ciego, con una muestra de 107 díadas madre-hijo. Los criterios de inclusión fueron madres primerizas sanas y bebés a término sin condiciones patológicas conocidas, con peso entre 2500 y 4000 gramos e indicación de lactancia materna exclusiva o predominante. En el grupo experimental las madres recibieron entrenamiento en la terapia de estimulación auditiva, táctil, visual y vestibular y lo aplicaron desde el nacimiento (primeras 24 horas de vida) hasta el final del seguimiento. El grupo control recibió solamente la atención estándar que incluyó la educación sobre los signos de alarma y las indicaciones básicas sobre la lactancia materna. Se midió la eficacia de la succión, el tipo de alimentación y el crecimiento neonatal en la semana 2 y la semana 5.

**Resultados.:**

Los bebés del grupo experimental comparados con el grupo control bebieron 2.02 cc más leche humana en un minuto de lactancia efectiva (*p*=0.002) en la segunda semana y 5.51 cc más en la quinta semana (*p*<0.0001); las madres registraron una mayor adherencia a la lactancia a las 5 semanas (*p*=0.025) y los bebés ganaron más peso: 8.35 gramos/día (*p*=0.009) y 4.19 gramos/día (*p*=0.008). El perímetro cefálico no presentó diferencias entre grupos, mientras que la diferencia en la talla fue estadísticamente significativa en la semana 5 (*p*=0.025).

**Conclusión.:**

La terapia de estimulación auditiva, táctil, visual y vestibular tiene un efecto positivo en la salud neonatal, promueve la succión eficaz, la lactancia materna exclusiva, atenúa la pérdida de peso y mejora el crecimiento neonatal.

**Objetivos.:**

Medir o efeito da terapia de estimulação auditiva, táctil, visual e vestibular na sucção eficaz, o modo de alimentação, o peso, tamanho e perímetro cefálico dos neonatos a término.

**Métodos.:**

Ensaio aleatório cego, com uma mostra de 107 díade mãe-filho. Os critérios de inclusão foram mães de primeira viagem saudáveis e bebês a término sem condições patológicas conhecidas, com peso entre 2500 e 4000 gramas e indicação de lactância materna exclusiva ou predominante. No grupo experimental as mães receberam treinamento na terapia de estimulação auditiva, táctil, visual e vestibular e a aplicaram desde o nascimento (primeiras 24 horas de vida) até o final do seguimento; o grupo de controle recebeu somente a atenção padrão que incluiu a educação sobre os sinais de alarme e as indicações básicas sobre a lactância materna. Se mediu a eficácia da sucção, o tipo de alimentação e o crescimento neonatal na 2ª semana e na 5ª semana.

**Resultados.:**

Os bebês do grupo experimental comparados com o grupo de controle beberam 2.02 cc mais leite humana num minuto de lactância efetiva (*p*=0.002) na segunda semana e 5.51 cc mais na quinta semana (*p*<0.0001); as mães registraram uma maior aderência à lactância às 5 semanas (*p*=0.025) e os bebês ganharam mais peso: 8.35 gramas/dia (*p*=0.009) e 4.19 gramas/dia (*p*=0.008). O perímetro cefálico não apresentou diferenças entre grupos, enquanto a diferença no tamanho foi estatisticamente significativa na 5ª semana (*p*=0.025).

**Conclusão.:**

A terapia de estimulação auditiva, táctil, visual e vestibular tem um efeito positivo na saúde neonatal, promove a sucção eficaz, a lactância materna exclusiva, atenua a perda de peso e melhora o crescimento neonatal.

## Introduction

Breast milk is the best food for newborns, yet there is low adherence to this feeding practice worldwide. In Colombia, 72.7% of newborns are breastfed, but only 36.1% of them continue to be breastfed at six months.(1) The breastfeeding cessation is influenced by several factors, including the experience of mothers who sometimes describe breastfeeding as difficult. This perception can be worse when they are young, first-time mothers, or have undergone cesarean section. Breastfeeding difficulties involve biological, psychological, and social factors(2) and occur in more than 70% of mothers during the first month of the child’s life.(3) Some physical difficulties in newborns reported are a poor suck or latch to the breast and drowsiness.(4) In the mother, the perception of an insufficient supply of milk is one of the predictors of exclusive breastfeeding cessation or the initiation of breast milk substitutes.(1) This kind of perception is associated with lactogenesis II, where there is an inverse relationship between the time to secrete milk and the infant’s effective sucking.(5) On average, lactogenesis II occurs 60 hours after birth but it may take longer. Primiparity is a risk factor strongly associated with delayed onset of lactogenesis II.(6) Therefore, it is very important to establish sucking effectiveness (SE) early, which is determined by the inter-suck breaks, the number of sucks, and the intraoral pressure.(7) SE is expressed in milliliters of breast milk taken per suckling, which increases spontaneously in the first 15 days of life to an average of 70 ml per feeding event. This volume depends on the intraoral pressure improvement;(8) if muscle strength increases, the number of sucks per burst and inter-suck breaks are reduced, and more milk is taken with less effort. Also, delayed lactogenesis II is associated with increased neonatal weight loss, insufficient weight gain, or jaundice. These after-effects often motivate health professionals to recommend breast milk substitutes to supplement infant feeding. Therefore, mothers should be able to stimulate effective sucking early to ensure optimal growth of the child.

Therapies such as the auditory, tactile, visual, and vestibular (ATVV) stimulation, proposed in the 1970s and modified in 1994,(9) have proven, throughout approximately three decades of research, to have favorable results in regulating behavioral states, the mother-child relationship, and the feeding behavior of preterm newborns who face great difficulty in regulating states of alertness due to prematurity.(10) The ATVV intervention has helped improve feeding progression, intraoral pressure, and the number of sucks in preterm infants,(11) and weight gain in full-term and preterm infants.(12) Therefore, these benefits can also be verified in full-term infants and produce positive results concerning the common breastfeeding difficulties experienced during the first month of life, a critical period that often defines breastfeeding success or failure. This is the first study in which mothers give ATVV therapy at home; previous studies have been conducted in hospitals or orphanages. Health professionals support breastfeeding initiation and maintenance; particularly, during counseling, they listen to mothers, help identify obstacles, and guide decision-making.(13) Nurses are in a privileged position to promote strategies that increase breastfeeding adherence. This study aims to measure the effect of ATVV stimulation therapy on breastfeeding (SE and infant-feeding mode), and neonatal growth (weight, height, and HC) of full-term infants.

## Method

Design. This article shows a secondary analysis from a larger study on the effect of infant stimulation on adaptation to birth, (14) a single-blinded randomized controlled trial study with two groups -experimental group (EG) and control group (CG)- and random allocation. The EG mothers were trained in ATVV therapy following the adapted Rice’s protocol(9) and received the standard care. The ATVV therapy involves using four sensory stimuli: auditory, with the mother’s voice humming a lullaby; tactile, with a sequential massage from less sensitive body areas to those with more nerve receptors; visual, with permanent eye-to-eye contact during therapy; and vestibular, with a few minutes of rocking in the mother’s arms. Previously trained mothers delivered the ATVV stimulation therapy twice a day, in the morning and at night, from the first 24 hours until the first two weeks after birth. From then on, the therapy was delivered once a day until the end of follow-ups. The CG mothers received standard care, which is education on warning signs, when to go back to the hospital, and basic recommendations on breastfeeding. A pediatrician, a research assistant, and two video analysts, blinded to group allocation, assessed SE, infant-feeding mode, and neonatal growth (weight, height, and HC). The data were taken at the Children’s University Hospital of San José Bogotá D.C., from July to November 2016. Only the results on the physiological adaptive mode “nutrition” are presented here using the same sample size from the main study.

Sample. Inclusion criteria were first-time mothers with no illnesses and breastfeeding intention and healthy full-term infants weighing between 2500 and 4000 grams with exclusive or predominant breastfeeding recommendation. Exclusion criteria were mothers under 18 years of age, mothers diagnosed with mental illness or cognitive deficit, hospitalization of the mothers or newborns, and breastfeeding cessation. The sample size was calculated for two independent groups, estimating a type I error of 0.04, a type II error of 0.01, and two groups to be compared (K=2). With these parameters, a sample size of 120 mother-child dyads was calculated, and the dyads were allocated randomly to CG or EG using a table with random numbers. The sampling method was consecutive and stratified with an equal allocation according to the mode of birth (i.e., vaginal delivery or Cesarean section). The percentage of participants lost to follow-up was 10.8% despite the measures taken by the research team. For the last follow-up, participants’ distribution was CG=53 and EG=54. To see the participant flow diagram, refer to the article entitled "Effect of infant stimulation on birth adjustment: a randomized trial.”(14)

Measurement. The variables measured were suckling effectiveness (SE), infant-feeding mode, and neonatal growth (weight, height, and HC). Analysis of video recordings by two independent analysts and the test weighing method (weighing the infant before and after breastfeeding), which can estimate milk intake from the third day of life using well-calibrated scales,(15) were used to assess SE. The SE ratio was created measuring variables described in the literature.(7) *Total time per feeding*: Time in minutes that the newborn takes from latching to voluntarily releasing the nipple. *Inter-suck break*: Time the newborn rests between suck bursts > 1.5 seconds. *Number of sucks*: Number of times the newborn sucks on the mother’s breast. *Suck burst*: A cluster of sucks, which differs from another by an inter-suck break. *Volume of breastmilk taken (cc)*: Difference in infant’s weight before and after breastfeeding, considering that 1 gram of weight is equivalent to 1 cc of milk taken. The SE indicator was calculated as follows:

$SE\;\frac{Volume\;of\;breasmil\;(cc)}{Time\;of\;Effective\;Suckling\;[TES]\;}$

Where

TES=Total time per feeding-Intersuck breaks (minutes)

Infant-feeding mode. The feeding mode the baby was breastfed in the last 24 hours was assessed and classified as exclusive breastfeeding or predominant breastfeeding. In exclusive breastfeeding, the infants received only breast milk from their mother or a wet nurse or expressed breast milk, and no other liquids or solids except for drops or syrups consisting of vitamins, mineral supplements, or medicines. On the other hand, in predominant breastfeeding, the infants are fed mainly on breast milk; however, the infants may also receive water and water-based drinks (i.e., teas, infusions); fruit juice, and Oral Rehydration Salts (ORS) solution.(16)

Growth. Using anthropometric measurements, the variables weight, height, and HC were was determined. Daily weight gain: This is the difference between the current weight and the birth weight, divided by the number of days of life at the time of measurement; it is measured in grams per day (g/day). Height gain: This is the difference between the current height and the height at birth, divided by the number of days of life at the time of measurement. Head circumference gain: This is the difference between the current HC and the HC at birth, divided by the number of days of life at the time of measurement. Height and HC were measured in centimeters per day (cm/day). Confounding variables such as gestational age (GA), Apgar test score, and the baby’s age at the time of assessment were measured. The GA was estimated using the New Ballard Score, and the Apgar score was determined using the Apgar test. Early initiation of breastfeeding is not a policy of the hospital. The data were obtained from participants’ medical records.

Data collection. The medical records of potential participants were reviewed to verify that the inclusion criteria were met. The dyads were recruited during the first 24 hours postpartum. The research was explained to the families, sociodemographic data was collected, and mothers’ breastfeeding intention was asked. The mother-child dyads were randomly allocated to EG and CG, and EG mothers were trained in ATVV stimulation therapy according to the protocol. Resources were provided to perform the therapy at home and audio-visual tools were shared with the participants (see video created by Lucy Marcela Vesga and María Mercedes Durán entitled “Teaching early auditory, tactile, visual and vestibular (ATVV) stimulation” posted on YouTube on May 28, 2019 at https://www.youtube.com/watch?v=AEYg6UaOAV4 and the audio lesson created by the same authors entitled “Step-by-step instructions” posted on YouTube on October 24, 2019 at https://www.youtube.com/watch?v=H6iIib42uCs). Dates for follow-up meetings at the hospital were agreed on. Birth anthropometric measurements were obtained from medical records. A pediatrician and a research assistant who were unaware of the random allocation of the groups made face-to-face follow-ups at weeks 2 and 5 to the mother-infant dyads. For these follow-ups, the infant had to have been breastfed 2 hours before the appointment to equalize the satiety level and wear only one diaper for taking the anthropometric measurements. Breastfeeding was videotaped, and the end of it was considered when the baby spontaneously let go of the mother’s breast, and she judged that the baby was finished. After breastfeeding, a research assistant weighed the infant wearing only a diaper on the same scale used for weighing the infant before the feeding. The mother was asked how she had breastfed the baby 24 hours earlier. Two video analysts followed a protocol to review each minute of the video recordings by sections, measuring criteria and optimizing the quality of the data obtained.

Data analysis. Data were analyzed using the SPSS 22.0 software. Concordance coefficients were used to ensure the quality of the data derived from the analysis of the video recordings. The data analysis followed two routes: one to compare between groups (CG and EG), and the other to compare within the groups at two different points in time (weeks 2 and 5). The effect size was calculated, and a linear regression model was created. The level of significance was set at *p* < 0.05.

Ethical issues. The study was approved by the hospital’s research ethics committee (Approval 066 June 2016) and the university’s research ethics committee (Approval 016-2016). Informed consents were signed by the mothers and by both parents approving the baby’s participation. This study was registered at the Australian New Zealand Clinical Trials Registry (ANZCTR) with registration number ACTRN1261717000449336 on March 27, 2017.

## Results

The analysis was performed with 107 mother-infant dyads (EG: 54 vs. CG: 53). Baseline data do not represent statistically significant differences between groups.

**Table 1:** Participants’ baseline characteristics

Characteristics	Experimental Group (*n*=54)	**Control Group (*n*=53)**	***p*-value**
Infants			
Gestational age (weeks); mean ± SD	38.9± 1.0	39±0.9	0.9*
Weight (g); mean ± SD	3077.3± 362.9	3152.7±361.4	0.3*
Height (cm); mean ± SD	50.7±1.8	50.3±1.4	0.2*
Head circumference (cm); mean ± SD	33.7±1.1	33.9±1.2	0.2*
Mothers			
Age (years); mean ± SD	25.2±6.2	24.3±4.2	0.9*
Occupation; *n* (%)			0.27**
Stay-at-home mom	18 (33.3)	15 (28.3)	
Employee	27 (50)	31 (58.4)	
Student	9 (16.6)	7(13.2)	
Breastfeeding intention; *n* (%)			0.062**
3 months	0 (0)	1 (1.8)	
6 months	3 (5.5)	6 (11.3)	
12 months	26 (48.1)	30 (56.6)	
>12 months	25 (46.2)	16(30.1)	

* Mann Whitney U test p-value; ** Chi-square test p-value

### Effect of ATVV stimulationg therapy on breastfeeding

Sucking Effectiveness (SE). Data quality was assessed by calculating Lin’s Concordance Correlation Coefficient (CCC). It ranges from -1 to 1, with perfect agreement at 1 and good agreement when it is greater than 0.7.(17) Lin’s CCC between the two analysts of the video recordings ranged from 0.903 to 0.996 for the first measurement of sucking parameters (number of sucks, number of bursts, inter-suck breaks), and it was 0.99 in all sucking parameters for the second measurement. 

**Table 2:** Effect of ATVV on Sucking Parameters

Time Sucking Parameters	Week 2	Week 2	Week 2	Week 5	Week 5	Week 5
Time Sucking Parameters	Control Group	Experimental Group	U Mann Whitney Test	Control Group	Experimental Group	U Mann Whitney Test
Time Sucking Parameters	*Mean*	*Mean*	***p*-value**	*Mean*	*Mean*	***p*-value**
*Burst*	79.31	73.12	0.393	81.28	75.75	0.305
*Number of sucks per burst*	9.47	10.15	0.282	9.87	9.71	0.486
Time of Effective Sucking (TES) (minutes)	9.35	9.02	0.715	9.94	8.94	0.152
Total time per feeding (minutes)	21.7	19	0.139	20.2	19.5	0.318

Table 3 shows the description of SE. There are no statistically significant differences between the EG and the CG. However, the results support the theory that ATVV stimulation promotes sucking development since there is a lower number of bursts, a higher number of sucks per burst, and a shorter sucking time in the EG than in the CG. This is a clinically meaningful result even though it is not statistically significant. These findings suggest that ATVV stimulation may increase muscle strength during sucking (not measured in this study), which allows for greater milk expression per minute. When comparing between groups the amount of breast milk expressed at week 2, it is observed that EG babies manage to express 2.02cc/TES more than CG babies (EG: 7.19 cc /TES vs. CG: 5.17 cc/TES, Mann-Whitney U-test p=0.002). At week 5, EG babies got 5.51 cc/TES more than CG babies (EG: 14.19 cc/TES vs. CG: 8.67 cc/TES, Mann-Whitney U-test *p*=0.002).

In the analysis of related samples, the Wilcoxon sign test showed statistically significant differences in both groups considering that time matures neonatal sucking ability. However, the benefits of ATVV stimulation are not unimportant, as seen in the regression model. A linear regression model was used and had an R-Squared of 0.336. Transformation of the dependent variable (SE) was necessary using the natural logarithm function to normalize the data distribution, a transformation that was verified using the Kolmogorov Smirnov test (p=0.200). Assessing the overall relevance of the model, its value p<0.001 allows us to affirm that the joint presence of the variables is relevant and, therefore, individual analysis of them is pertinent. In this sense, belonging to the EG and being a girl increase the natural logarithm of SE, while predominant breastfeeding decreases it. SE at week 2, weight gain, and neonatal age *directly correlate with SE at week 5*. These results confirm the importance of stimulating effective sucking early, as it can influence the feeding process in the long term; the use of breast milk substitutes can hinder SE. Other variables such as mode of birth, GA, birth weight, Apgar test score, use of epidural analgesia, or breastfeeding intention were removed from the model because they were not statistically significant (Table 3).

**Table 3:** Linear Regression Model for Sucking Effectiveness (SE) at Week 5

Model	Non-standardized coefficients	Non-standardized coefficients	Standardized coefficients	*t*	***p*-value**
Model	B	Standard error	Beta	*t*	***p*-value**
Constant term	1.546	0.820		1.886	0.062
Comparison group	0.356	0.133	0.234	2.679	0.009
Sex	0.256	0.131	0.168	1.954	0.054
Weight gain	0.025	0.009	0.265	2.824	0.006
Infant-feeding mode	-0.282	0.168	-0.147	-1.683	0.095
Sucking effectiveness at week 2	0.031	0.016	0.172	1.874	0.064
Infant’s age	-0.017	0.022	-0.066	-0.789	0.432

### Infant-feeding mode

Regarding the feeding mode, a higher frequency of exclusive breastfeeding was observed in the EG dyads, a difference that is only statistically significant at the follow-up measurement at week 5. The achievement of effective sucking in the EG infants possibly improved mothers’ confidence in their ability to breastfeed, and for this reason, they return to exclusive breastfeeding (Table 4). 

**Table 4:** Effect of ATVV on Infant-feeding Mode

Infant-feeding mode by follow-up measurement	Group	Group	Chi-square
Infant-feeding mode by follow-up measurement	Control (*n*=53)	Experimental (*n*=54)	***p*-value**
Week 2			
Exclusive breastfeeding	41(77.3)	47(87)	0.19
Predominant breastfeeding	12 (22.7)	7(13)	0.19
Week 5			
Exclusive breastfeeding	38(71.7)	48 (88.9)	0.025
Predominant breastfeeding	15 (28.3)	6 (11.1)	0.025

### Effect of ATVV on infants’ growth

Weight, height, and HC variables were age-adjusted during statistical analysis to control bias. Table 5 shows a weight gain of 8.35 g/day in the EG at week 2 (Cohen’s d = 0.54) compared to the CG. It is observed that weight loss slows down as compared to the CG. Meanwhile, at week 5, the difference between the groups is 4.19 g/day (Cohen’s d = 0.53), which suggests a dose-dependent effect of ATVV therapy.

**Table 5:** Effect of ATVV on infants’ growth

Variable	Group	Group	Difference	Cohen’s d	Mann-Whitney U
Variable	Control (*n*=53)	Experimental (*n*=54)	Difference	Cohen’s d	***p*-value**
Weight (g/day); mean					
Week 2	4.34	12.69	8.35	0.54	0.009
Week 5	29.10	33.29	4.19	0.53	0.008
Height (cm/day); mean					
Week 2	0.08	0.10	0.02	0.3	0.337
Week 5	0.08	0.11	0.03	0.4	0.025
Head circumference (cm/day); mean					
Week 2	0.12	0.10	0.02	0.3	0.515
Week 5	0.086	0.10	0.014	0.78	0.041

## Discussion

No research on SE was found in full-term infants, though four studies evaluating SE in preterm infants were found. In these studies, babies had 23 to 33 weeks of GA, received 1 to 3 sessions of ATVV therapy, and researchers measured variables such as the transition from tube to oral feeding, time from the start to the end of the feeding, and milk-intake. Regarding oral feeding achievement, there are studies that did not prove the effectiveness of ATVV therapy (11,12) and studies that found a 4-day advantage of EG infants in achieving complete oral feeding, a difference statistically significant, (18,19) and increased oral intake during the first ten days of ATTV therapy(18) In our study, EG babies expressed more breast milk in both follow-up measurements than CG babies, and this difference was statistically significant. Compared to the number of sucks, these studies showed statistically significant differences between the groups, before and after the intervention; however this effect disappears when an analysis of variance is performed.(18)

The same occurs with the amount of milk taken and the time spent in feeding. These studies found sucking differences between the groups at 7 days post-intervention, which were a greater number of sucks, a greater number of sucks per burst, and a greater sucking pressure of preterm infants in the EG. These differences disappeared after 14 days of therapy. In our study, a difference not statistically significant was observed. Medoff-Cooper et al.(11) interpreted such results as the effect of neurological maturation and evolution of the infants and suggested that ATTV stimulation therapy may favor the earlier achievement of neurobehavioral organization and, thus, sucking maturity.(12)

Regarding the infant-feeding mode, no known studies precede this study and show the effect of ATVV therapy. One explanation we propose for this effect is that, by improving SE more quickly, lactogenesis II is accelerated;(5) this may increase the mother’s confidence in her ability to breastfeed (6,20) and control the weight loss. In terms of the effect of ATVV therapy on weight gain, some studies differ mainly in the participants enrolled, who were hospitalized, preterm infants. The studies also differ in terms of the number of therapy sessions.(18,19) These differences affect comparability because preterm infants are under regulated temperature conditions, have diseases associated with a larger amount of energy expenditure, are fed on strict schedules, and have a controlled caloric intake. Furthermore, in some cases, fortified breast milk is used to increase nutritional intake of preterm infants; all of this is made to favor weight gain. However, research has found a positive effect of ATVV therapy on neonatal weight gain (18,19) and suggests a dose-response effect.(18)

In healthy full-term infants, there is a previous study using ATVV. The infants received two daily sessions, five days a week for four weeks, by a trained professional. They were admitted to the study at 2 weeks of age and were followed up to 6 months. All of the infants lived in an orphanage. The EG infants showed a daily weight gain that doubled the CG infants in the first four weeks. The limited socio-affective expressions and the use of breast milk substitutes may explain the size of the effect. The results agree that there is a favorable effect of ATVV stimulation therapy on neonatal weight gain.(12) Among the most solid hypotheses that explain the effect is that vagal system stimulation, activated by tactile stimulation, allows for a greater release of hormones such as insulin, thereby improving the gastric absorption of food.(21) However, confounding variables such as human milk exposure to tobacco metabolites and environmental pollution have not been controlled in any research.(22) Regarding height and HC, our results are similar to those reported by Kim et al., who found a difference of 0.87 cm in height in EG infants versus CG infants, and HC 1.78 cm larger in EG infants.(23) HC is an indicator of brain growth, and some studies have found that HC did not predict cognitive or fine motor scores but greater gross motor skills in boys, and it is affected by emotions, the environment,(24) and other factors. Therefore, it is understandable that a difference exists in the effect size between both studies. Another study tested the effect of ATVV in premature infants between 29 and 34 GA, who received two daily sessions of stimulation therapy during their hospitalization. The researchers found a difference between the groups in terms of height, though they found no difference in HC.(24)

## Conclusion

This study shows that the ATVV stimulation therapy increases SE in full-term infants from week 2 onwards and perhaps increases adherence to exclusive breastfeeding, thereby becoming a technique to be used during the most challenging period of breastfeeding, as mothers can be trained quickly, and there are tools designed to support providing this therapy at home. Another important result of this research is that providing ATVV therapy at home reduces the weight loss in the first days and favors the neonatal growth expressed in weight gain, height, and HC. This is a significant result, as babies with low weight, borderline weight, or risk factors may benefit. Therefore, the beneficial effects of ATVV stimulation therapy can contribute to the health of healthy newborns and also to those full-term infants who are vulnerable and at nutritional risk.

Routine use of ATVV stimulation therapy in full-term infants during the first few weeks at home may help improve SE and, thus, the mother’s confidence in her ability to breastfeed. This, in turn, could improve adherence to exclusive breastfeeding. Future studies should evaluate the effect of the therapy on longer follow-ups and control confounding variables that were beyond the scope of this research, such as early initiation of breastfeeding and exposure to tobacco toxins or environmental pollutants. The results are important for the clinical practice of health professionals, as they allow for evidence-based, effective, and presumably low-cost strategies in inpatient and outpatient settings. In clinical practice, stimulation therapy should replace the use of other stimuli such as cold (removing clothes, passing a wet cloth) to address the alertness difficulties that hinder strong and continuous sucking and the correct execution of the learned techniques for breastfeeding, as stimuli like these can cause stress in babies and have harmful repercussions.

Limitations. This research had budget limitations, as it did not receive any funding. In addition, there was a significant loss of participants, which required great logistical effort to continue the study. Despite this, at the end of the fieldwork, the loss of participants was a little over 10%. This study did not control variables such as exposure to environmental pollution or tobacco metabolites, intake of other liquids, or time of exclusive breastfeeding that may influence neonatal weight gain.
